# Supplementary material for: Expression of the Arabidopsis thaliana immune receptor EFR in Medicago truncatula reduces infection by a root pathogenic bacterium, but not nitrogen‐fixing rhizobial symbiosis
Source: Plant Biotechnol J. 2018 Sep 13;17(3):569–79. doi: 10.1111/pbi.12999 (PMC6381793; doi:10.1111/pbi.12999)
Supplement: Supplementary file 1 — Figure S1 EFR expression does not affect development of M. truncatula. Figure S2 Alignment of elf18 peptide sequences. Figure S3 M. truncatula responds to flg22 peptide. Figure S4 MAPK activation and marker gene induction is induced upon elf18 treatment in EFR‐expressing lines. Figure S5 Transgenic EFR‐Medicago roots and nodules accumulate EFR. Figure S6 EFR expression in M. truncatula provides quantitative resistance against the pathogen R. solanacearum. Figure S7 Dose‐dependent ROS response of M. truncatula roots from EFR‐expressing lines 26‐8 and 18‐1 to elf18 peptide. [file PBI-17-569-s001.pdf]

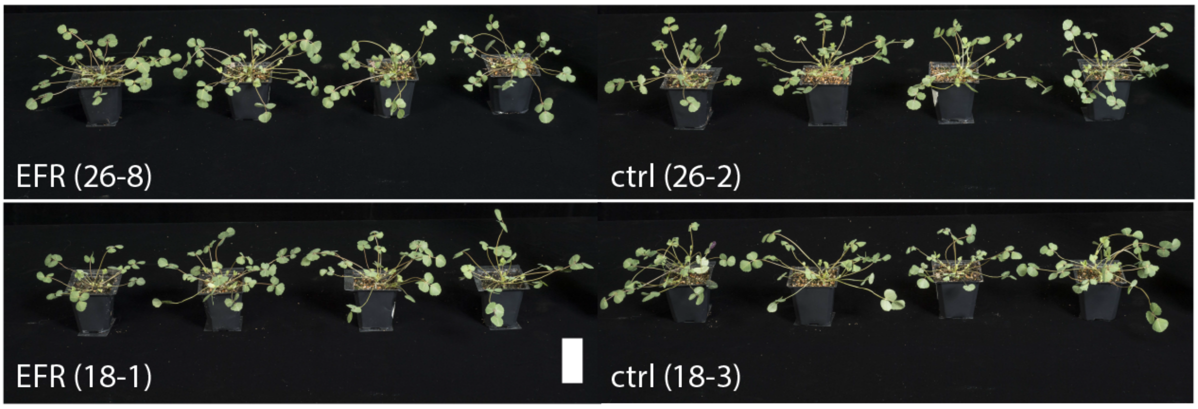

Fig S1. *EFR* expression does not affect development of *M. truncatula*. Phenotype of two independent stable *EFR*-expressing *M. truncatula* lines, 26-8 and 18-1, and their null segregant control lines 26-2 and 18-3, respectively. White scale bar, 5 cm.

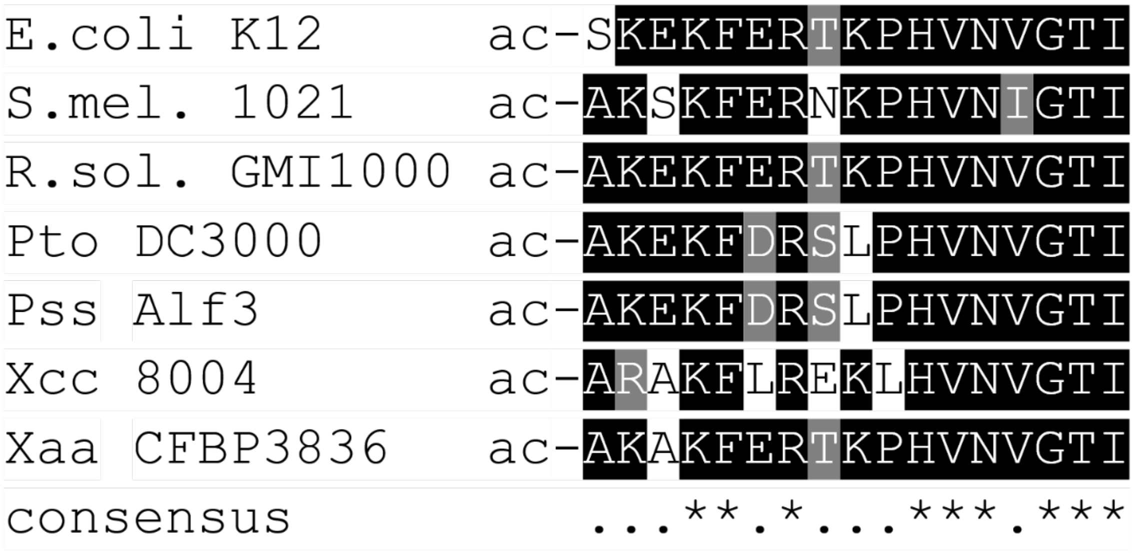

Fig S2. Alignment of elf18 peptide sequences. Peptide sequences are displayed with N-terminal acetylation (ac-) from following species: *Xanthomonas alfalfae* subsp. *alfalfae* CFBP3836, *X. campestris* pv. *campestris* 8004, *S. meliloti* 1021, *Escherichia coli* K12, *R. solanacearum* GMI1000, *Pseudomonas syringae* pv. *syringae* ALF3, *P. syringae* pv. *tomato* DC3000. Elf18 peptide sequences belong to seven groups with different eliciting activity according to Lacombe *et al.* 2010. Multiple sequence alignment has been created with Boxshade v3.21. Shadings indicate different degrees of conservation. Asterisk (*) in consensus indicates identity across all sequences.

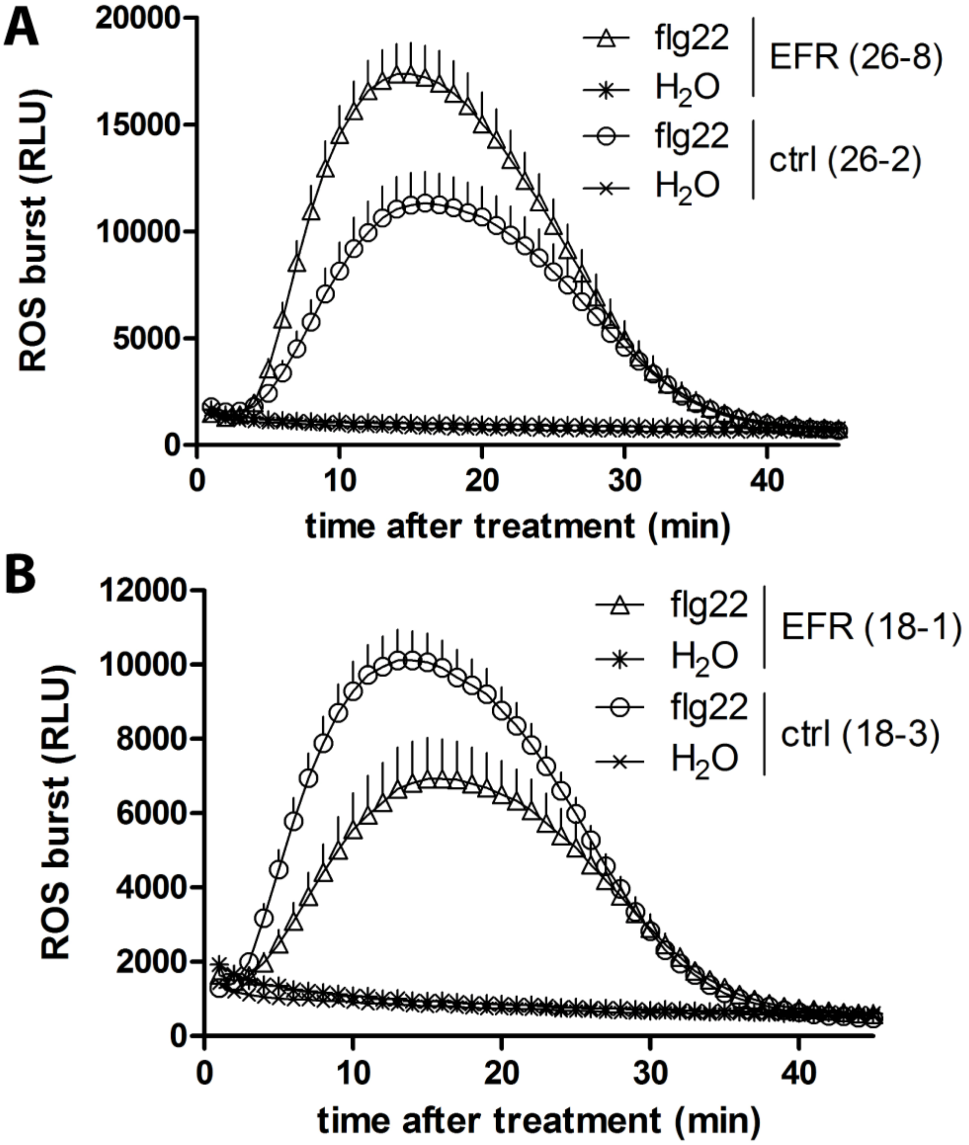

Fig S3. ***M. truncatula*** **responds to flg22 peptide.** ROS burst was monitored in root segments from line 26-8 and 26-2 (A) and from line 18-1 and 18-3 (B) after application of 100 nM flg22 peptide and displayed as relative light units (RLU). Values are means ± standard error (*n*=8). Experiment was performed twice.

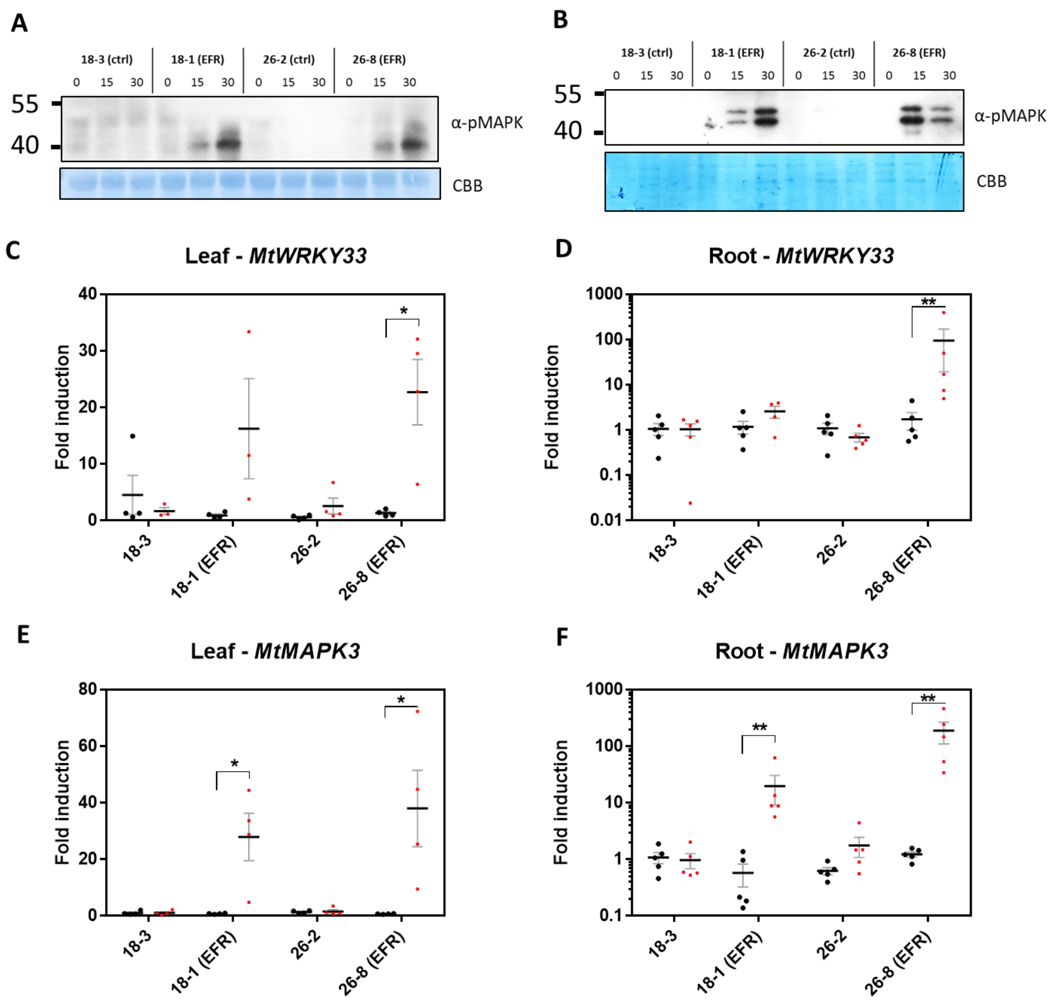

Fig S4. MAPK activation and marker gene induction is induced upon elf18 treatment in *EFR*-expressing lines . (A,B) Leaves (A) or roots (B) of *M. truncatula* were treated with 1 µM elf18 for 0, 15 or 30 minutes. Immunoblot analysis was performed using anti-phosho-p44/42 MAPK antibody. CBB staining was used as a loading control. This experiment was repeated three times with similar results. (C-F) Quantitative RT-PCR analysis of immune-related genes after 1h treatment with 1 µM elf18 (red symbols) or water (black symbols). *MtWRKY33* (C,D) and *MtMAPK3* transcripts (E,F) were analysed independently in leaf (C,E) and root tissues (D,F). Values represented are relative to water treatment at time 0 of each experiment. At least three experiments were performed with similar results. Means and SEM are shown by horizontal and vertical bars, respectively. Stars indicate significant differences determined by Wilcoxon test (* p≤0.05, ** p≤0.01).

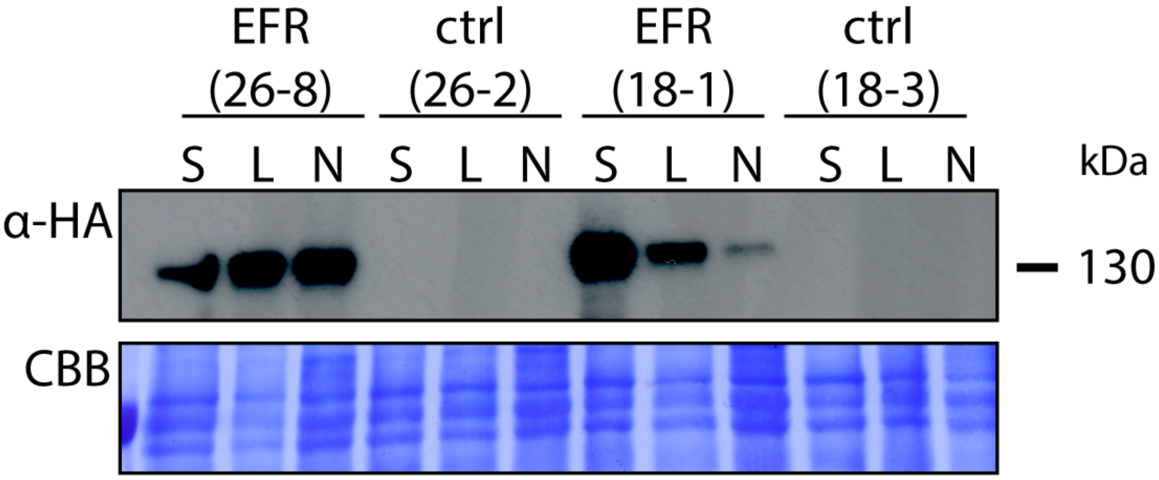

Fig S5. Transgenic EFR-*Medicago* roots and nodules accumulate EFR. Accumulation of EFR can be detected in stem root (S), lateral roots (L) and nodules (N) by western blot using α-HA antibody. Root material and nodules were harvested after inoculation with *Sm*1021-*lacZ* at 28 dpi. Membrane was stained with Coomassie Brilliant Blue (CBB) as loading control. Experiment was performed twice.

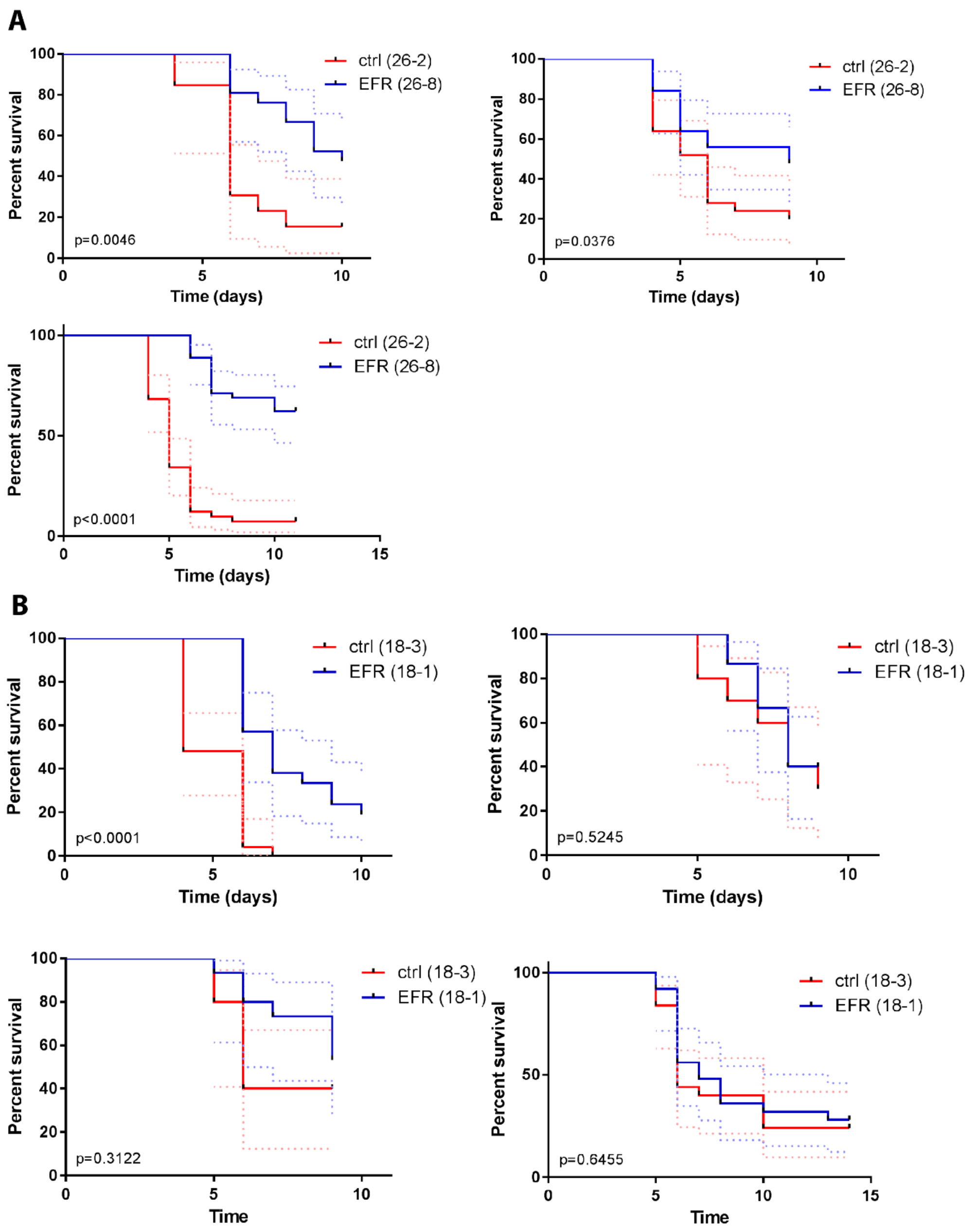

Fig S6. *EFR* expression in *M. truncatula* provides quantitative resistance against the pathogen *R. solanacearum*. (A) *M. truncatula* line expressing *EFR* 26-8 and control line 26-2 (A) and line expressing *EFR* 18-1 and control line 18-3 (B) were infected with *R. solanacearum* GMI1000 and disease symptoms assessed daily. Survival rate is displayed over time and statistical analysis performed with Mantel-Cox test (n=25). Dashed lines represent 95% confidence interval.

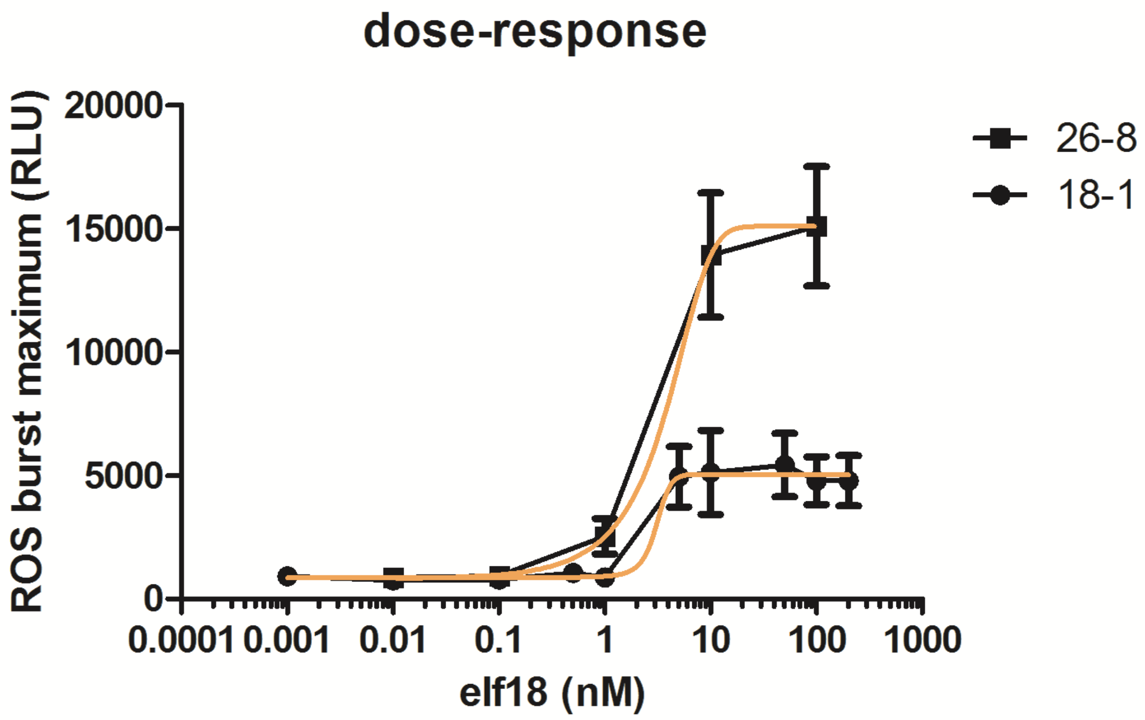

Fig S7. Dose-dependent ROS response of *M. truncatula* roots from *EFR*-expressing lines 26-8 and 18-1 to elf18 peptide. ROS burst maximum (displayed as relative light units) was monitored in root segments of lines 26-8 and 18-1 and plotted against elf18 peptide concentration. Orange line was calculated by the sigmoidal non-linear fit function in GraphPad Prism 5. Values are means ± standard error (*n*=8). Experiment was performed three times.
